# Effects of chemical modifications on hemoglobin’s toxicity towards human cardiac myocytes

**DOI:** 10.3389/fmolb.2025.1648209

**Published:** 2025-08-28

**Authors:** Sirsendu Jana, Haley Garbus-Grant, Tigist Kassa, Abdu I. Alayash

**Affiliations:** Laboratory of Biochemistry and Vascular Biology, Center for Biologic Evaluation and Research, Food and Drug Administration, Silver Spring, MD, United States

**Keywords:** hemoglobin-based oxygen carriers (HBOCs), redox reactions, mitochondrial fractions, cardiac AC16, cytotoxicity

## Abstract

**Background:**

Hemoglobin-based oxygen carriers (HBOCs) also known as blood substitutes were developed by chemical or genetic alterations of cell-free human or bovine Hbs to prolong the circulation time of Hb and to improve its ability to unload oxygen. However, toxicity and safety issues led to the termination of several clinical trials. The most persistent observation was the development of cardiac lesions after transfusion of some HBOCs in animal models. Oxidation of HBOCs in circulation, subsequent heme release and cellular uptake are thought to play an important role in the overall toxicity of HBOCs.

**Methods:**

We examined the effects of different redox states, ferrous (Fe^+2^), ferric (Fe^+3^) and ferryl (Fe^+4^) of four different HBOCs on cardiomyocyte integrity and mitochondrial respiration. The HBOC formulations used in this study were two-human derived and two bovine-derived molecules. We analyzed cellular and subcellular impacts of these forms including mitochondrial electron transport chain (ETC.) complexes individually by measuring the enzymatic activities of Complex I, Complex II-III, and Complex IV.

**Results:**

The ferrous, and ferric forms of these HBOCs generally induced minimum lactate dehydrogenase (LDH) release from human cardiac myocytes (AC16). Meanwhile higher oxidation state, ferryl forms of all HBOCs generated substantial cell injury as measured by LDH levels. We examined the effects of these redox forms of HBOCs and their ability to impair bioenergetic function of cultured AC16 cells. The ferrous forms of HBOCs did not cause measurable impairment of mitochondrial ETC functions, whereas ferric non-functional versions of all the HBOCs caused a significant loss of Complex IV activity but not Complex I or II-III in those cardiac cell lines. On the other hand, complex I, II-III and IV activities were completely blunted by the ferryl forms of HBOCs.

**Conclusion:**

This study for the first time investigated the impact of different chemical modifications on the redox activities of HBOCs towards mitochondrial complexes in cardiac myocytes. Higher oxidation ferryl states once formed trigger cellular and subcellular changes in cardiac myocytes. Our findings on the impact of HBOC redox states on mitochondrial function may therefore inform future design of alternative molecular entities to ensure safety and minimize toxicity.

## Introduction

In the late 1990s, research and development of hemoglobin (Hb)-based oxygen carriers (HBOCs), or “blood substitutes,” reached the highest levels of activity with several products reaching phase 3 pivotal clinical trials. Unfortunately, clinical evaluation of HBOCs in humans has been disappointing, with toxicity leading to the discontinuation of their development. The most reported adverse events in human trials included hypertension, cerebrovascular events, renal dysfunction, pancreatic and liver enzyme elevation, and myocardial events (Natanson et al., 2008). The reaction of cell-free Hb with nitric oxide generated by the vascular endothelial lining of blood vessels leading to vasoconstriction and elevation in blood pressure is the most likely mechanism underlying these effects (Gould et al., 1998; Olson et al., 2004). The second proposed mechanism is the rapid unload of oxygen by cell-free Hb, leading to vasoconstriction as an autoregulatory response (Winslow, 2013). In recent years experimental evidence emerged to support a third mechanism which focuses on heme-mediated side reactions that ultimately lead to tissue toxicity (Alayash, 2004; Alayash, 1999).

Heme-mediated redox reactions of Hb occur in red blood cells (RBCs), despite the presence of several reducing and antioxidant mechanisms within these cells. These reactions are accelerated in disease states and during room storage temperature of blood in blood banks. In both sickle cell and aged RBCs these reactions yield highly reactive species of Hb which can target band 3 proteins and lead to structural changes in RBCs (Strader et al., 2020; Kassa et al., 2024).

Outside the RBCs, free Hb undergoes rapid conversion to highly oxidized species at an accelerated pace, leading to tissue damage if uncontrolled. The heme prosthetic group in the ferrous (HbFe^2+^) state reversibly binds oxygen, leading to oxidation of the heme iron to the ferric form (HbFe^3+^) in a process known as autoxidation (Alayash and Wilson, 2022). The slow autoxidation of the oxygen-carrying HbFe^2+^→HbFe^3+^, a non-oxygen carrying ferric (metHb), is a single electron process which leads to the production of superoxide ions (O2^•−^) that rapidly dismutate to form hydrogen peroxide (H_2_O_2_). The resulting H_2_O_2_ triggers a cascade of oxidative reactions with either ferrous or ferric Hb. These reactions lead to the generation of a potent oxidizing ferryl Hb (HbFe^4+^) species, as well as a secondary radical from the reaction between H_2_O_2_ and metHb (Kassa et al., 2024). However, unlike classical peroxidases, Hb is unable to harness this radical (^•^HbFe^4+^), which then migrates to a group of amino acids in an area on the protein known as the oxidation “hotspot”, primarily targeting βCys93 site (Alayash and Wilson, 2022).

Chemical and genetic alterations of Hb to generate cell-free HBOCs have been found in some cases to lead to accelerated oxidative reactions and formation of a persistent ferryl species (Silaghi-Dumitrescu et al., 2007). Ferryl Hb once formed is known to be a highly reactive molecule that impacts cellular and subcellular components including the mitochondria. Mitochondria are central to cellular bioenergetics generating most ATPs as the “powerhouse,” through a tightly controlled oxidative phosphorylation (OXPHOS) and play a critical balancing act in maintaining oxygen homeostasis (Jana and Alayash, 2025). Mild to moderate alteration in cellular bioenergetics is seen in many chronic diseases and therefore represents a potential therapeutic strategy. Mitochondrial bioenergetic analyses have not been explored extensively in the context of HBOC cytotoxicity. Recently however, our group and others have shown that heme and cell-free Hb-mediated disruption of mitochondrial bioenergetic function in different cell types (Cardenes et al., 2014; Higdon et al., 2012; Chintagari et al., 2016; Kassa et al., 2015). We found that exposure of E10 cells to ferryl Hb resulted in a drop in hyperpolarized cell percentage over untreated control, thus indicating a significant mitochondrial depolarization or compromised mitochondrial respiration. We also found heme-mediated loss of mitochondrial trans-membrane potential (Δψm) following exposure of E10 cells to hemin (10 µM) for 24 h (Chintagari et al., 2016).

In an isolated rat heart Langendorff perfusion system we found that perfusion with a ferric form of crosslinked Hb caused significant impairment of mitochondrial cytochrome c oxidase. We also observed many fused mitochondria that maintained their round shape following hypoxic perfusion, but with partial loss of cristae membrane was observed following perfusion with ferric Hb (Edmondson et al., 2019).

Here, we incubated 4 different chemically modified HBOCs (human and bovine) in different redox (ferrous, ferric and ferryl) states with human-derived cardiomyocytes (AC16), and we monitored mitochondrial respiration of cells and the enzymatic activities of their respective complexes. We noted very little differences in the ETC activities among the ferrous forms of HBOCs vs. their native unmodified Hbs. However, ferric, and most notably ferryl forms of HBOCs were more damaging and more greatly initiated cellular and subcellular changes.

## Materials and methods

### Proteins and chemicals

Human and bovine Hbs were purified as previously described using anion and cation chromatography (Meng et al., 2018). BvHb and HbA represent unmodified native bovine and human Hbs controls respectively The HBOC (DCLHb) (Diaspirin cross linked Hb (αXLHb) was a gift from the US Army. All other crosslinked HBOCs were gifts from the respective manufacturers through Material Transfer Agreements (MTAs) and were used with no further purification. The HBOCs O-R-PolyHbA0 (Hemolink), PolyBvHb (Hemopure) were studied for human use and PolyBvHb (Oxyglobin) for veterinary use. Chemicals and reagents were purchased from Sigma Aldrich (Saint Louis, MO) or Fisher Scientific (Pittsburgh, PA) unless otherwise indicated. Gases were purchased from Roberts Oxygen Company, Inc (Rockville, Maryland). H_2_O_2_ (30% w/w) was purchased from Sigma. Dilute solutions of H_2_O_2_ were prepared fresh daily from a stock solution by making appropriate dilution in deionized water and kept on ice. The concentration of H_2_O_2_ was determined spectrophotometrically at 240 nm using a molar extinction coefficient of 43.6 M-^1^cm-^1^. Buffer solutions were prepared by mixing solid monobasic and dibasic potassium phosphate dissolved in deionized water and the pH was adjusted accordingly. All experiments carried out with Hbs or HBOCs were conducted as per the Center for Biologics Evaluation & Research (CBER), FDA guidelines.

### Ferric Hb preparation

Stock solutions (1.5 mM) of ferrous Hb were treated with slight molar excesses of K3 [Fe(CN)6] to generate ferric Hb. Removal of K3 [Fe(CN)6] was accomplished using a column containing Sephadex G-25 media (Sigma). Concentrations of the MetHb were determined by the OD at 630 nm (ε = 3.78 mM-^1^).

### Ferryl Hb preparation

Ferrous Hbs (500 μM) were incubated with H_2_O_2_ (5 mM (10x) for 5 min and followed by the addition of catalase (200 units/mL) that was reacted for 1 min to ensure the complete removal of H_2_O_2_. Ferryl Hb formation was detected using a previously described method by adding 2 mM sodium sulfide (Na_2_S) to the reaction mixture, which transforms the ferryl Hb to a sulfhemoglobin (sulfHb). Fresh stock solutions of Na_2_S were prepared for each experiment. The levels of ferrylHb were estimated using an extinction coefficient of sulfHb (24 mM^−1^ cm^−1^) at 620 nm (Berzofsky et al., 1971).

### Cell culture

AC16 cells derived from primary adult ventricular tissue were obtained from ATCC. These cells can be induced to differentiate into mature cardiomyocytes. Cells were grown in DMEM F12 media supplemented with 12.5% FBS and 1% penicillin/streptomycin.

### HBOC Treatments

For exposure to Hbs and HBOCS, AC16 cells were seeded in 6 well plates with about 3 × 10^5^ cells/well for LDH assay or in 75 cm^2^ flasks with about 5 × 10^6^ cells/flasks for mitochondrial ETC assay experiments. On the day of exposure, cells with approximately 80% confluency were kept for at least 6 h in a serum-free DMEM F12 media before Hb or HBOC exposure. Each Hb or HBOC were diluted to a final concentration of 60 µM in serum-free DMEM F12 for exposure to cells (18 h). Approximately 1 mL media were collected from each well to assess LDH release, then cells were rinsed with PBS.

### Cell cytotoxicity assay

Cellular cytotoxicity was measured in cultured AC16 cells following exposure to different HBOCs and Hbs using an Invitrogen™ CyQUANT™ LDH Cytotoxicity Assay Kit (Cat #C20300, Thermo Fisher Scientific). LDH activity was quantified by a coupled enzymatic reaction in which LDH catalyzed the conversion of lactate to pyruvate via reduction of NAD + to monitor cytotoxicity. Briefly, after the incubation, 50 μL of sample medium was transferred to a 96-well flat bottom plate in triplicate wells. For the LDH Positive Control, 50 μL aliquot of LDH Positive Control was transferred into triplicate wells. Then 50 μL of reaction mixture was added to each sample well followed by gentle mixing and incubated at 37 °C for 30 min in the dark. The assays were stopped by adding the Stop Solution, and then absorbance was measured using a microplate reader at 490 and 680 nm. The level of formazan formation is directly proportional to the amount of LDH released into the medium, which is indicative of cytotoxicity.

### Isolation of cardiac mitochondria

Mitochondrial fraction was isolated from AC16 cells by differential centrifugation after completion of Hb and HBOC treatments. Cultured cells were thoroughly washed with PBS (2 times) to ensure complete removal of the residual Hb proteins. and then homogenized using a Teflon glass homogenizer (10–12 strokes) in 1 mL mitochondria isolation buffer containing sucrose (290 mM), KH2PO4 (5 mM), MgCl2 (5 mM), HEPES (2 mM), EGTA (1 mM) and BSA (0.2%), and all subsequent steps of the preparation were performed on ice. Homogenate was centrifuged at 1500 *g* for 10 min at 4 °C. Following centrifugation, the supernatant was collected and further centrifuged at 12000 *g* for 10 min at 4 °C to obtain the mitochondrial pellet. The pellet was resuspended in same buffer and centrifuged again at 12000x g for 10 min at 4 °C. The pellet was washed with a BSA-free mitochondria isolation buffer. Finally, the pellet was resuspended in BSA free mitochondria isolation buffer and aliquoted in desired volume. Total protein (mg/mL) was determined using Pierce BCA Protein Assay Reagent (Cat # 23227, Thermo Fisher, Waltham, MA, United States). Isolated mitochondrial fractions were kept on ice and immediately used for mitochondrial ETC complex assays. An aliquot of the mitochondrial suspension (25 μg of protein) was utilized to measure for each complex activity.

### Complex I assay

The enzymatic activity of NADH-dehydrogenase (Complex I) was assayed by using ferricyanide as the electron acceptor (Jana et al., 2017). The assay was carried out at 30 °C in a reaction system containing 0.17 mM NADH, 0.6 mM potassium ferricyanide, 0.1% (v/v) Triton-X 100 in 50 mM phosphate buffer, pH 7.4. The rate of oxidation of NADH was monitored by the decrease in absorbance at 340 nm after the addition of mitochondrial suspension to the sample cuvette (Jana et al., 2011; Khan et al., 2005).

### Complex II-III assay

Succinate supported reduction of ferricytochrome c to ferrocytochrome c at 550 nm was monitored to measure the activity of mitochondrial complex II–III (succinate–cytochrome c reductase) following the method by Jana et al. (2011). The reaction was initiated by adding the mitochondrial suspension (20 μg) to the sample cuvette. The absorbance change at 550 nm was monitored for a period of 3 min. The assay was repeated with antimycin A (10 μM) and the enzyme activity was calculated by subtracting the antimycin A sensitive rate from overall rate and expressed as μmoles oxidized cytochrome c reduced/min/mg protein (Jana et al., 2011).

### Complex IV assay

The activity of complex IV was assayed following the oxidation of reduced cytochrome c (ferrocytochrome c) at 550 nm in 10 mM phosphate buffer, pH 7.4 at room temperature (Khan et al., 2005). K-ferricyanide (1 mM) was added to oxidize ferrocytochrome c in the blank cuvette and the reaction was initiated in the sample cuvette by the addition of mitochondrial suspension. The activity of the enzyme was calculated from the first order rate constant and the concentration of reduced cytochrome c in the sample cuvette as published earlier (Khan et al., 2005; Wharton and Tzagoloff, 1967).

Interference of any residual Hb bound to mitochondria was monitored by using appropriate blanks, and blank values were subsequently subtracted from respective assays.

### Statistical analysis

All values are expressed as mean ± SEM. Values from two treatment groups were compared using paired Student’s t-test using GraphPad prism software. A p-value of <0.05 was considered as statistically significant.

## Results

### Biochemical and molecular characteristics of HBOCs

In this investigation, we selected four (4) HBOCs that have gone through extensive evaluation in humans and in animals in Europe, USA/or Canada (Estep, 2025) (Table 1). Two of these HBOCs were derived from human Hb (stroma free) (SFH) in the case of HemeAssist (DCLHb) (Baxter) or from a highly purified HbA_0_ in the case of Hemolink (O-poly-R-HbA_0_) (Hemosol). DCLHb (also known sometimes as DBBF) is generated by the reaction of the reagent, *Bis*(3.5-dibromosalicyl)-fumarate to form a link between two lysines (Lys99 α1 and Lys99 α2) (Meng et al., 2018). DCLHb like other HBOCs gone through extensive purifications and viral inactivation with minimum residual RBC’s contaminants. DCLHb was extensively tested in various preclinical and clinical studies. However, in phase 3 clinical trials, patients treated with DCLHb had significantly higher mortality rates than control groups (Sloan et al., 1999).

**TABLE 1:** Biochemical and molecular characteristics of HBOCs.

Products^b^	Company	Chemical Modifier(s)	Molecular weight (KDa)	P_50_ (mmHg)
HemAssist/ DCLHb (αXLHb)	Baxter	Bis(3,5-dibromosalicyl)-fumarate 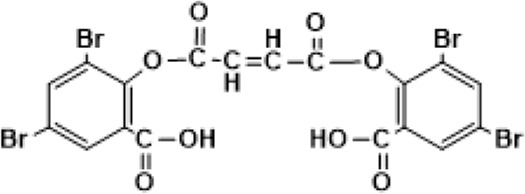	64	30
Hemolink (*O*-R-PolyHbA_0_)	Hemosol Inc.	O-raffinose 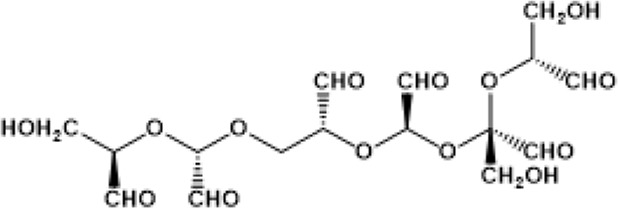	<64: <5%; 64-500: >90%; >500: <3%	34
Hemopure (PolyBvHb)	Biopure Corp.	Glutaraldehyde 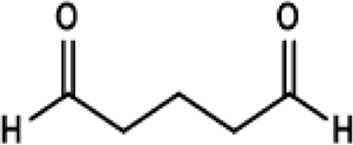	<64: < 2.5%; Average M.Wt. 250	38
Oxyglobin	Biopure Corp.	Glutaraldehyde 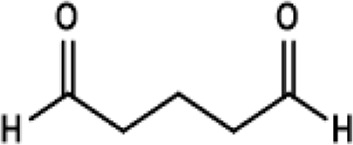	<64: < ∼30-35%; Average M.Wt. 200	38

Functional and structural properties of commonly investigated HBOCs (commercially and non-commercial nomenclature is used here to describe the nature of chemical/genetic modification(s). Structural representations of the reagent (s) used to chemically modify human and bovine hemoglobin to produce HBOCs as well as molecular sized and their oxygen binding properties (P50) (Silverman and Weiskopf, 2009; Meng et al., 2018).

Hemolink (*O*-poly-R-HbA_0_) (manufactured by a Canadian company, Hemosol) is a polymerized Hb with an unusual oxygen characteristic; non cooperative and non-sigmoidal oxygen equilibrium curves (OECs) were the result of reacting activated sugar, *O*-raffinose with HbA_0_. Proposed sites of intramolecular crosslinking (i.e., β1Lys82, β2Lys82, and β1Val1) by manufacturers were not found to be the predominant sites of crosslinking within the central cavity. Mass spectrometric analysis showed that intermolecular crosslinking with *O*-raffinose no discernible site of amino acids modifications except for β93Cys and α104Cy (Boykins et al., 2005). Hemolink has been tested in a series of clinical trials including in patients undergoing elective coronary artery bypass graft surgery (Cheng et al., 2004).

Both Hemopure (PolyBvHb) and Oxyglobin were manufactured by Biopure Inc., for human and veterinary use respectively using the same non-specific polymerizing reagent, glutaraldehyde. The degree of polymerization in the human product is more extensive than its animal counterpart. The residual tetramer in Hemopure is less than 2.5% as opposed to 30%–35% in Oxyglobin (Buehler et al., 2005). Hemopure was initially evaluated in phase 2 in acute hemorrhage and later in a pivotal trail phase 3 prehospital trauma patients with acute anemia. Hemopure was approved in South Africa in adult’s patients with acute anemia (Estep, 2025).

### Oxygen binding characteristics of HBOCs

Table 2 lists some of the biochemical and functional characteristics of HBOCs included in this study and are compared with their native unmodified Hbs. Both DCLHb and *O*-poly-R-HbA_0_ showed oxygen binding properties close to that of an RBC, with a P_5o_ (oxygen affinity of Hb when half saturated) around 29–32 mmHg. The OEC curve for *O*-polyHbA_0_ is reported to be right shifted with a P_50_ value of 32 mmHg and appeared to be less sigmoidal in shape compared to its precursor, HbA_0_. One striking feature of *O*-polyHbA_0_ is the none-cooperativity of its OEC with an n = 1.0 instead of being a round 2.5–2.7 (Meng et al., 2018). The OECs of both PolyBvHb, and Oxyglobin are less sigmoidal and reported to lack saturation at the normal physiological range of 90–100 mmHg. However, using complete OECs generated by Adair constants allowed for accurate determination of their P_50_s (Alayash et al., 2001).

**TABLE 2:** Oxygen binding, Autoxidation, and Peroxide-mediated Oxidation Parameters.

Hemoglobin	P_50_ (mmHg)	*n*_50_ ^a^	Bohr effect	K_autox_ ^b^ h^-1^	Sulfheme,^c^ (Hb:H_2_O_2_, 1:1), μM	K_heme loss_^*d*^, h^-1^
HbA	11.28 ± 0.40	2.61 ±0.08	1.77 ± 0.11	0.043 ± 0.001	10.90 ± 0.56	10.39 ± 0.1
αXLHb	29.10 ± 0.27	2.05 ±0.10	0.78 ± 0.10	0.081 ± 0.003	18.80 ± 0.21	7.30 ± 0.05
*O*-R-PolyHbA_0_	32.40 ± 1.29	1.00 ± 0.05	0.32 ± 0.03	0.130 ± 0.010	20.40 ± 0.70	7.30 ±0.04
BvHb	20.76 ±1.97	2.66 ± 0.10	1.38 ± 0.30	0.120 ± 0.010	15.87 ± 0.31	12.10 ± 0.72
PolyBvHb	34.28 ± 0.77	1.75 ± 0.04	0.38 ± 0.03	0.220 ± 0.020	24.00 ± 1.13	3.47 ± 0.91
Oxyglobin	34.59 ± 0.67	1.71 ± 0.08	0.49 ± 0.02	0.210 ± 0.020	22.60 ± 0.57	4.50 ±0.95

^a^ Oxygen equilibrium curves (OECs) for human Hb-based and bovine Hb-based HBOCs, were carried out in the Hemox Analyzer. The experiments were carried out with 70 μM Hb in 10 mM phosphate buffer, pH 7.4, containing 0.1 M NaCl, anti-foaming agent, and the Hayashi enzymatic reducing system at 37 C. Measurements of the Bohr effect of human Hb-based and bovine Hb-based HBOCs, and the P50 was measured at pH ranging from 5.5 to 9 in 0.1 M bis–Tris buffer or Tris buffer with 0.1 M NaCl at temperature of 37 °C.

^b^ The value for autoxidation (K_auto_ h^-1^) is the rate constant for the spontaneous oxidation of hemoglobins measured at 37 °C in 50 mM phosphate buffer, PH, 7.4.

^c^ Sulfhemoglobin (sulfheme) was measured by reacting the ferryl hemoglobin with sodium sulfite (Na2S) and the resultant sulfheme was measured at 620 nm using published extinction coefficients (Berzofsky et al., 1971). Data taken from Bioconjug Chem (Meng et al., 2018).

Table 2 also lists some of the physiological affectors of the oxygen affinity of Hb such as the Bohr effects (i.e., changes in oxygen affinity over a wide range of acidic and alkaline pHs) (Meng et al., 2018). The Bohr coefficients derived from the slopes (ΔlogP50/ΔpH) for native human and bovine Hb were 1.77 and 1.38 H per tetramer, respectively, in agreement with previously reported values (Alayash et al., 2001) whereas for the chemically/genetically modified Hbs, the values calculated were as low as 0.23–0.78 H (Table 2). A 50%–80% reduction in the proton-linked oxygen release from HBOCs may reflect a direct or indirect blockage of some Bohr amino acid residues caused by these modifications (Meng et al., 2018).

The spontaneous oxidation (autoxidation) of the human HBOCs, DCLHb and *O*-poly-R-HbA_0_ are ∼ 2-3-fold higher than that of their native, HbA respectively whereas bovine Hb-based products have a ∼2-fold increase in the rate of their autoxidation than that of unmodified bovine Hb. Reactions of the modified Hbs with H_2_O_2_ (1:1) were compared in their susceptibility to chemically induced oxidation and subsequent oxidative changes and found to be generally 1.4–1.8 higher rates than their native unmodified proteins (Table 2).

### HBOC cytotoxicity in cardiac cells

**FIGURE 1:**
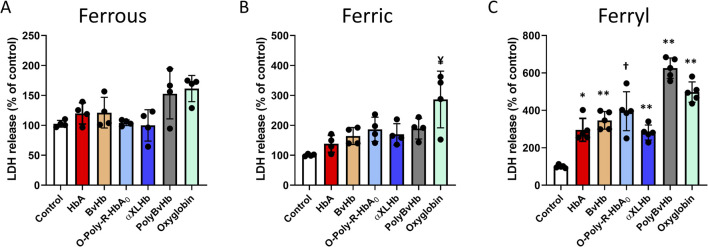
Oxidized Hbs and HBOCs cause LDH release from cardiac myocytes. Cardiac AC16 cells were grown in 6 well plates for LDH assay and treated overnight (12 h) with either **(A)** ferrous or, **(B)** ferric or, **(C)** ferryl forms of various Hb or HBOCs (60 μM). Following incubation, LDH activity was measured photometrically in the media as described in the method section. Values are presented as % of respective untreated controls and vertical error bars represent standard deviation. Differences between treated groups and respective untreated controls were compared using unpaired Student’s t-test (N = 4). P values are calculated against respective untreated controls. ¥P < 0.0078 vs. ferric control, *P < 0.001, **P < 0.0001, † P < 0.0002 vs. ferryl control.

In this study, we used an immortalized cardiac cell line (AC16) derived from adult ventricular muscles to examine the cytotoxicity of human and bovine Hbs and HBOCs with different chemical modifications and different redox states of these proteins (ferrous, ferric and ferryl). Ferrous and ferric forms of Hbs and HBOCs used in this study did not cause any noticeable LDH release with the exception with ferric form of Oxyglobin, which caused a moderate increase in LDH in the media indicating some level of cytotoxicity (Figures 1A,B). Notably, both the human and bovine Hbs and the HBOCs when oxidized to ferryl forms caused a significant LDH release indicating a substantial cell injury (Figure 1C). Again, bovine derived HBOCs PolyBvHb and Oxyglobin seem to induce substantial LDH release from these myocytes.

### HBOCs cause mitochondrial bioenergetic impairment

**FIGURE 2:**
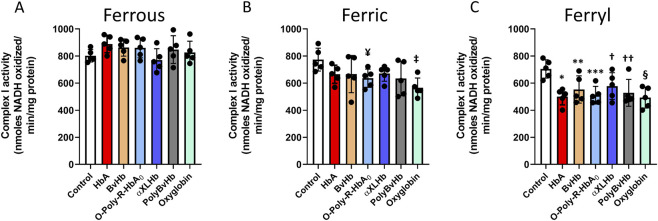
Oxidized Hbs and HBOCs cause Complex I inhibition in cardiac AC16 cells. Cardiac AC16 cells were grown in 75 cm^2^ flasks and treated overnight (12 h) with either **(A)** ferrous or **(B)** ferric or, **(C)** ferryl forms of various Hb or HBOCs (60 μM). Following incubation, mitochondrial fractions were isolated from AC16 cells as described in the method section. Complex I enzymatic activity was measured in isolated mitochondrial fractions by photometric assay and Statistical significance between means was calculated by paired Student’s t-test. *P < 0.05 vs. respective untreated controls (N = 5). ¥ P < 0.022, ‡ P < 0.003 vs. ferric control; *P < 0.001, **P < 0.023, ***P < 0.0017, † P < 0.045, †† P < 0.011, § P < 0.0017 vs. ferryl control.

To understand the full extent of cellular and subcellular impact of the redox forms of Hb and HBOCs, we examined the effects of different redox forms of Hbs and the HBOCs on isolated mitochondrial fractions from cultured AC16 cells following treatments with the Hb molecules. We analyzed individual respiratory complex activities of the mitochondrial ETC obtained from the mitochondrial fractions. Figure 2 shows activities of complex I in AC16 cells following exposure to ferrous (A), ferric (B) and ferryl (C) forms of the HBOCs. None of the ferrous forms of Hbs/HBOCs used in this study had any effect on myocytes (Figure 2A). Similarly, ferric HBOCs were mostly non-inhibitory on complex I but ferric *O*-polyHbA_0_ and ferric Oxyglobin showed marginal inhibition of complex I activity (Figure 2B). In contrast, all the Hbs and HBOCs used in this study caused a significant loss of complex I activity when they were fully oxidized to their ferryl state (Figure 2C).

**FIGURE 3:**
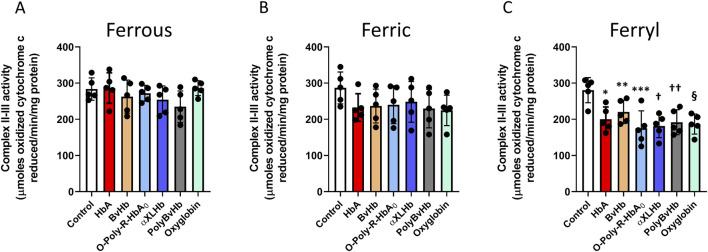
Oxidized Hbs and HBOCs cause Complex II-III inhibition in cardiac AC16 cells. Cardiac AC16 cells were grown in 75 cm^2^ flasks and treated overnight (12 h) with either **(A)** ferrous or, **(B)** ferric or, **(C)** ferryl forms of various Hb or HBOCs (60 μM). Following incubation, mitochondrial fractions were isolated from AC16 cells as described in the method section. Complex II-III enzymatic activity was measured in isolated mitochondrial fractions by photometric assay and Statistical significance between means was calculated by paired Student’s t-test. *P < 0.05 vs. respective untreated controls (N = 5). *P < 0.006, **P < 0.022, ***P < 0.004, † P < 0.0016, †† P < 0.0037, § P < 0.0015 vs. ferryl control.

Next, we examined the impact of these Hbs on complex II-III activities in those mitochondrial fractions. We did not find any change in Complex II-III with either ferrous or ferric forms of the HBOCs, although ferric HBOCs showed a marginal but non-significant loss of activities (Figures 3A,B). Ferryl states of all the Hbs and HBOC molecules caused a significant inhibition of complex II-III activities in AC16 cells similar to complex I inhibition seen (Figure 3C).

**FIGURE 4:**
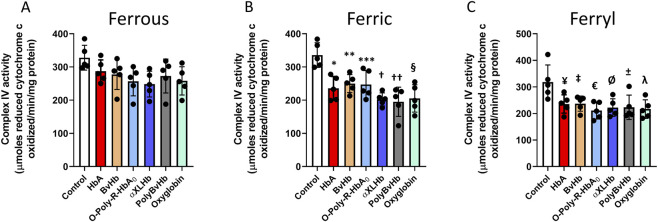
Oxidized Hbs and HBOCs cause Complex IV inhibition in cardiac AC16 cells. Cardiac AC16 cells were grown in 75 cm^2^ flasks and treated overnight (12 h) with either **(A)** ferrous or, **(B)** ferric or, **(C)** ferryl forms of various Hb or HBOCs (60 μM). Following incubation, mitochondrial fractions were isolated from AC16 cells as described in the method section. Complex IV enzymatic activity was measured in isolated mitochondrial fractions by photometric assay and Statistical significance between means was calculated by paired Student’s t-test. *P < 0.05 vs. respective untreated controls (N = 5). *P < 0.0025, **P < 0.0031, *** P < 0.0078, † P < 0.0001, †† P < 0.0006, § P < 0.0008 vs. ferric control; ¥P < 0.036, ‡ P,0.033, €P < 0.01, ØP < 0.018, ±P < 0.028, λP < 0.015 vs. ferryl control.

Finally, we analyzed complex IV activities in the Hb and HBOC treated AC16 cells. As seen with complex I and II-III, ferrous HBOCs did not cause any noticeable damage to the complex IV. Surprisingly, all the ferric forms caused a modest inhibition of complex IV indicating a susceptibility to heme mediated damage (Figure 4B). As seen earlier, all the Hbs and HBOCs also caused a strong inhibition of complex IV activity indicating a consistent inhibitory potential of these highly reactive molecules (Figure 4C).

## Discussion

Because of the confidentiality in disclosing the nature of HBOC toxicity by manufacturers and regulatory authorities, very limited information on their side effects during human clinical trials were made in the open literature. Recently we were able to compare side by side biochemical and biophysical characteristics of all HBOCs that undergone clinical evaluation in variety of clinical settings after these HBOCs were made available to our group (Meng et al., 2018). In a follow up publication by Jahr et al. (2025), a correlation between these unique features reported by Meng et al., and their clinical outcome were carried out recently to specifically draw some general lessons that will hopefully enable the design of a safe and effective product. Based on this publication, clinical trials, and clinical outcomes were added to the biophysical and biochemical characteristics of the various products that enabled the formation of more definitive conclusions (Jahr et al., 2025).

A recent analysis of the incidence of myocardial infarctions (MI) in patients infused with 5 clinically tested HBOCs (4 out the 5 HBOCs were used in the current study) that focused on pharmacokinetic and biochemical parameters to identify correlations that are suggestive of cause-and-effect. There were positive correlations between MI incidence and HBOC dose, size, intravascular half-life, and area under the plasma concentration (total exposure of blood and endothelium to HBOCs *versus* time curve (AUC). Furthermore, MI incidence was positively correlated with initial rates of HBOC autoxidation, oxidation by nitric oxide, and AUCs estimated for these HBOC oxidation products. These observations imply that increased MI risk after HBOC infusion is due to intravascular reactions which exacerbate oxidative stress (Estep, 2025). The correlation between HBOC infusion, oxidative events and enhanced MI incidence may be the most rigorous proof to date of the hypothesis that oxidative stress increases MI risk (Estep, 2025).

In this study we examined the impact of different HBOC oxidative states (oxy/ferrous, ferric and ferryl) on cultured human cardiac cells. Specifically, we focused on the role of these species in cellular injury and mitochondrial respiration, specifically the correlation between HBOCs own oxidative reactions and consequent impact on cellular and subcellular components of cardiac myocytes. To understand potential mechanisms of toxicity of the HBOCs on human cardiac cells, we used AC16 cells which are possibly the only commercially available cardiac cell line of human origin derived from primary human ventricular tissue and can be induced to differentiate into cardiomyocytes. As shown in Table 1, autoxidation rates, ferryl levels as well as the rates of heme release from the ferric forms of these HBOCs, it is quite possible that increases in the ferrylHb activities play a key role in changes seen at the cellular and subcellular (ETC.) levels as described here. Generally, higher rates of autoxidation in human and bovine Hbs were matched by higher rates of oxidative modifications and ferrylHb formation. As reported in this table, chemically modified human and bovine Hbs autoxidize at rates 1.7–3.0-fold higher than their native unmodified Hbs, likely due to the sit-specific modification(s) on these proteins. Ferryl heme activities were also higher in the modified human and bovine Hbs, some 1.4 to 1.87-fold higher than their respective unmodified Hbs. It is interesting to note that the reduction in heme released from the oxidized forms of these HBOCs (due likely) to their specific chemistry of each HBOC was reduced by almost 1.4-fold.

We have previously shown that ferric Hb loses heme at rates substantially higher than ferryl Hb. This was also supported by a higher expression of heme oxygenase-1 (HO-1) when ferric Hb was added to cultured lung alveolar squamous epithelial cells (E10) (Kassa et al., 2016). However, ferryl Hb once formed can be more damaging to mitochondrial respiration in cardiac muscle cells than ferrous or ferric as evidenced by the data presented in this study. Although, relative contributions of different oxidative mechanisms in a specific tissue can be greatly influenced by the physiological status and function of the tissue (e.g., brain, heart, lungs etc.) (Granger and Kvietys, 2015), however, in a previous study using lung epithelial cells we observed a similar phenomenon where ferryl Hb exposure caused a robust translocation of HO-1 to mitochondria with a stronger impairment of mitochondrial respiration and loss of mitochondrial transmembrane potential compared to ferric Hb (Chintagari et al., 2016).

Lactate dehydrogenase (LDH), is an enzyme found in almost all tissues involves in cell energy production which can also be used to identify location and severity of tissue damage was used in our experiments as a reliable marker for HBOC’s mediated cytotoxicity. As shown in Figure 1 when myocytes are incubated with the ferrous form of HBOCs, there was little or no change in LDH levels with human derived HBOCs as opposed to slight or moderate increase in LDH levels released by myocytes in the presence of bovine HBOCs. The incubation of myocytes with the ferric forms of human and bovine derived HBOCs led to almost double the LDH release, particularly more so with bovine HBOCs. Using the ferryl species of these HBOCs we noticed substantial increase in cell injury and LDH release to as high as almost 600% (6 times) over untreated cells in the case of bovine derived HBOCs. Interesting to note that both Oxyglobin and Hemopure autoxidize at higher rates than their native Hb and human derived HBOCs and these higher rates and ferryl formation correlated with higher levels of LDH injury in myocytes.

The enhanced oxidation of HBOCs in plasma to methemoglobin has been previously noted in clinical trials (Sprung et al., 2002; Olofsson et al., 2006) despite human capacity to reduce plasma methemoglobin (Vandegriff et al., 1997). However, this reductive capacity may, however, be compromised or overwhelmed in patients with pre-existing endothelial dysfunction at higher HBOC doses (Biro, 2012). Spontaneous oxidation of heme iron within HBOCs in the presence of air (autoxidation) or in the presence of oxidants (oxidation) compromise the ability of HBOCs to deliver oxygen to tissues. In animal models (Lee et al., 1995) and in some clinical trials (Fitzgerald et al., 2011) metHb accumulation up to 30%–40% of total Hb were reported to comprise tissue oxygenation.

It has been known for some time that oxidative stress is correlated with increased MI risk (Wang and Kang, 2020). This is not surprising since the incidence of MI is highly correlated with the initial rate of HBOC autoxidation. The correlation between HBOC infusion, oxidative events and enhanced MI incidence is consistent with the notion that oxidative stress increases MI risk and HBOCs play a critical role in these events (Estep, 2025).

Mitochondria are known to play a critical role in cardiac energy output and are therefore critically important in energy-demanding cardiac muscle (Dai and Rabinovitch, 2009). The oxidation of several biological fuels e.g., NADH, pyruvate or succinate, is accomplished via the mitochondrial ETC that provides the energy required (Lesnefsky et al., 2001). Here, we investigated the biological significance of altered bioenergetics as a possible mechanism of cardiotoxicity in HBOCs using human-derived cardiomyocytes. None of the ferrous Hbs and HBOCs had noticeable effect on any of the respiratory chain complexes of the mitochondrial ETC. However, we found that the ferric forms of the Hbs and HBOCs caused mild inhibition on each ETC complex.

In a previous study, we evaluated myocardial performance and biochemical alterations in rat left ventricle (LV) in response to perfusion with a specific HBOC (DCLHb) following a brief hypoxic episode (Edmondson et al., 2019). LV performance recovery was enhanced following ferrous DCLHb infusion, indicating a cardioprotective effects possibly due to its ability to adequately oxygenate tissues during perfusion. In contrast, ferric DCLHb infusion caused heart lesion formation, which was partially reversed by ascorbate (a mild reducing agent) co-infusion, emphasizing the oxidative toxicity associated with ferric ions. This study showed that heme rapidly released by ferric Hb can also lead to oxidative radical species production and iron accumulation which was directly associated with diminished LV performance with an extensive damage in myocardial mitochondrial ultrastructure (Edmondson et al., 2019).

In the current study, we also observed that mitochondrial respiration was impacted by a mechanism involving impairment of mitochondrial ETC function especially the cytochrome c oxidase (complex IV) activity in agreement with our *in vivo* studies (Edmondson et al., 2019). It is generally accepted that cardiac contractility is strongly dependent on mitochondria, and that depletion of ATP is the main factor involved in myocardial dysfunction (Wal et al., 2023; Neubauer, 2007; Gupta et al., 2012). We have seen earlier that ferric Hbs can in fact facilitate uncoupling leading to upregulation of oxygen consumption in various cultured mammalian cells (Chintagari et al., 2016; Jana et al., 2017). A recent investigation showed that ferric Hb is the primary driver of cell-free Hb-induced lung microvascular mitochondrial dysfunction. Activation of the mitochondrial permeability transition (mPTP) and the release of mtDNA are a feature of ferric cell-free Hb mediated injury (Riedmann et al., 2025). Heme released from oxidatively unstable Hb can also cause activation of TLR4. Activated TLR4 and carbon monoxide (CO) are both known to cause the uncoupling of mitochondrial respiration (Kaczara et al., 2015; Kaczara et al., 2016). In contrast, ferryl forms of both human and bovine Hbs and their HBOC analogs were significantly damaging to all mitochondrial ETC complexes, further indicating the high reactivity and toxicity of the ferryl ions. Apart from the direct oxidative toxicity of the ferryl ions, many other secondary processes like heme and iron accumulation, oxidation of lipids and proteins can all contribute to the inhibition of bioenergetic functions and a general cellular toxicity (Edmondson et al., 2019).

The molecular basis of toxicity mediated by the ferric/ferryl hemes towards cellular systems and especially their effect on the bioenergetics of the mitochondria is not fully understood. We have previously shown the impact of ferric/ferryl forms on heart tissues (Edmondson et al., 2019) and in cultured endothelial/epithelial cells through possibly an electron transfer pathway from these redox active molecules in target cells (Kassa et al., 2015). We have also shown in our earlier study that ferric Hb shows high degree of heme release than ferryl Hb with concomitant HO-1 expression which can possibly be a mechanism behind ferric Hb/HBOCs mediated complex IV inactivation seen in this study (Kassa et al., 2016). We and other studies have shown HO-1 translocation to mitochondria under different stress conditions, indicating an interesting link between HO-1 overexpression and mitochondrial dysfunction (Bindu et al., 2015; Slebos et al., 2007; Kassa et al., 2015). Mitochondrial accumulation of HO-1 can lead to localized generation of toxic levels of CO upon degradation of the heme, thus leading to complex IV inhibition (Song et al., 2006). It is also possible that HO-1 translocation to mitochondria can be a compensatory protective effect of the HO-1 protein by scavenging extra heme load (Bindu et al., 2015; Slebos et al., 2007).

Several antioxidative strategies have been designed recently to target Hb-mediated oxidative pathways to control these reactions, specifically by the ferryl heme. These included the use of ferryl specific antioxidants, such as caffeic acid (Kassa et al., 2021), curcumin (Hicks et al., 2024), (−)-epigallocatechin gallate (EGCG) (Jia and Alayash, 2008), in addition to re-engineering of more oxidatively stable Hbs.

Apart from the direct oxidative toxicity of the ferryl ions, many other secondary processes like heme and iron accumulation, oxidation of lipids and proteins can all contribute to the inhibition of bioenergetic functions and a general cellular toxicity (Edmondson et al., 2019).

In summary, we showed in a simple cell culture system that a correlation exists between HBOC’s redox states and in particular higher oxidation ferryl heme and cellular and subcellular changes including changes in the mitochondria which may explain the reported cellular changes leading to cardiac lesion. It remains unclear however, the full extent of the molecular basis of HBOC-mediated mitochondrial dysfunction in the hearts. This remains as one limitation of this study. It follows therefore that any protective strategie(s) should include some design elements that minimize Hb’s oxidative reactions to control/reduce the toxicity of HBOC formulations (Bian and Chang, 2015). Finally, identifying how HBOCs may impact patient redox status and endothelial function would improve patient selection (Estep, 2025). In sum, understanding the mechanisms of HBOC toxicity will allow for the design of safer blood substitutes and help identify patient populations that are most likely to benefit from HBOC administration.

## Data Availability

The original contributions presented in the study are included in the article/supplementary material, further inquiries can be directed to the corresponding author.
